# A Rare Presentation of AKI: Gastric Infiltration of the Bladder Wall

**DOI:** 10.1155/2011/387649

**Published:** 2011-07-28

**Authors:** Sandipani Sandilya, Ladan Golestaneh

**Affiliations:** Montefiore Medical Center, 111 East 210th Street, Bronx, NY 10467, USA

## Abstract

We describe the case of a man who presented with back pain and acute kidney injury and was found to have bilateral ureteral obstruction, which initially corrected with ureteral stents. Imaging studies showed thickening of the bladder. Shortly thereafter, he developed obstructive jaundice, pancreatitis, recurrence of renal failure, and was diagnosed with advanced gastric cancer after a laparotomy revealed peritoneal carcinomatosis. The patient deteriorated rapidly after diagnosis. While peritoneal carcinomatosis, ureteral metastases, and extrinsic ureteral compression have been recognized in gastric cancer, obstructive renal failure due to tumor infiltration of the bladder wall is seldom described. We present this case as an unusual cause of acute renal failure and presentation of gastric cancer.

## 1. Introduction

Acute Kidney Injury (AKI) due to bilateral ureteral obstruction in older patients typically raises concerns of abdominal, retroperitoneal, or pelvic neoplasms. Malignancies cause hydronephrosis by compression, by direct extension, by lymph nodes, or by retroperitoneal fibrosis. It is rare for hydronephrosis, as a result of obstruction at the level of the ureteral bladder inlet, and back pain to be the presenting symptoms of gastric cancer [1]. We describe the case of a man with advanced gastric cancer who initially presented with back pain and renal failure due to ureteral obstruction and hydronephrosis from bladder pathology. 

## 2. Case Report

### 2.1. Clinical History and Initial Laboratory Data

A 48-year-old Hispanic man with no significant past medical history started experiencing abdominal and back pain worse with exertion. He self-medicated with ibuprofen and sought medical attention after two weeks as the pain became progressively worse. At presentation, he was febrile, with nausea and vomiting, had some dysuria, postvoid dripping, and constipation. He denied any hematuria or rectal bleeding. Initial examination was unremarkable apart from right costovertebral angle (CVA) tenderness. The initial lab values are presented in Table 1. Baseline values were unavailable.

### 2.2. Imaging

**Figure 1:**
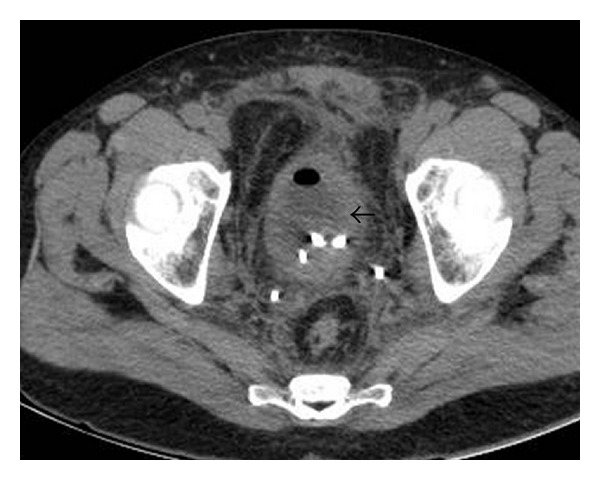
A thickened bladder wall (arrowhead) with stranding of the mesenteric fat, status post-JJ stents. Despite the collapsed bladder, the wall thickening is clearly visible.

The chest X-ray was unremarkable, and the abdominal X-ray showed evidence of ileus. A CT showed a thickened bladder wall with infiltration of the adjacent fat, bilateral hydronephrosis and hydroureters, hepatomegaly, and trace perihepatic ascites (Figure 1). 

A foley was inserted with minimal urine output. Bilateral JJ ureteral stents were placed two days after admission, and his renal function improved. Shortly thereafter, he started spiking fevers and was started on vancomycin and zosyn. Cultures were negative, and the patient was discharged on oral augmentin for a few more days with planned outpatient followup. 

He presented again to the same institution three weeks after discharge, complaining of nausea, vomiting, poor appetite, and abdominal pain. His admission laboratory results are presented in Table 2.

He was initially treated for a urinary tract infection. Ultrasound showed gall bladder sludge, but no stones, and a normal common bile duct (CBD). His HIDA scan showed total retention of Tech 99 in the liver suggestive of CBD obstruction. A CT scan showed pancreatitis, hepatomegaly with perihepatic and perisplenic ascites, esophageal and gastric varices, and intact bilateral JJ stents. A repeat ultrasound showed portal vein thrombosis. A liver biopsy showed histological changes consistent with large bile duct obstruction and focal cholestasis, without evidence of autoimmune hepatitis. Endoscopy, performed after coffee ground emesis, showed three nonbleeding gastric ulcers and duodenitis in the bulb. He was subsequently transferred to our institution. 

**Figure 2:**
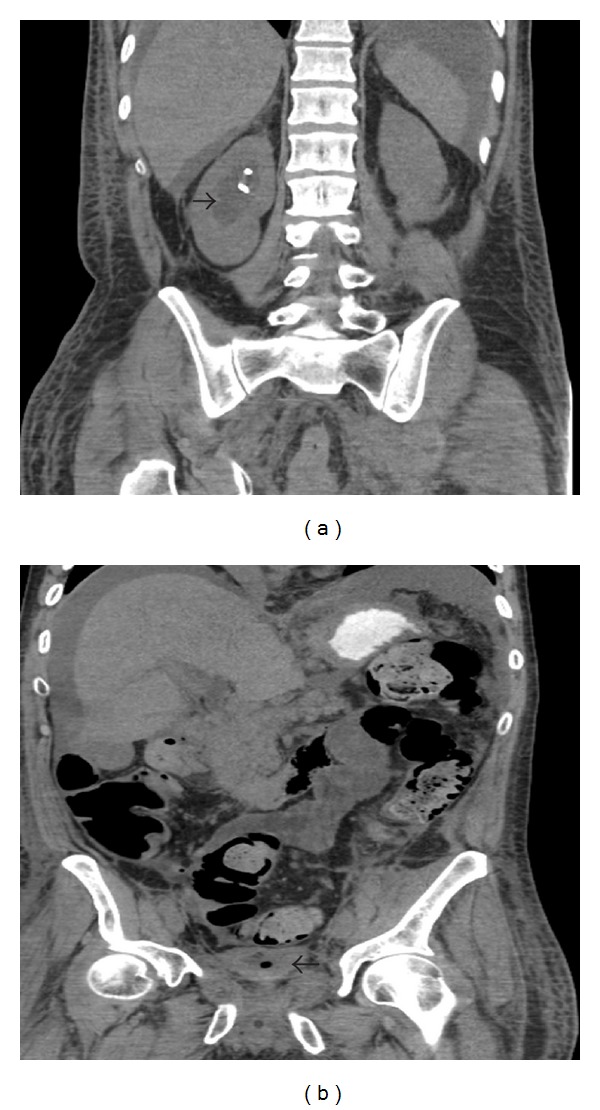
Images from our institution showing a thickened bladder wall (arrowhead on the right) and right-sided nephrosis (arrowhead on the left).

At the time of transfer, he was experiencing abdominal pain and drowsiness. He denied any other symptoms. Examination was significant for jaundice and diffuse abdominal tenderness without rebound tenderness or guarding. For the investigation and treatment of his obstructive jaundice, an ERCP was performed, revealing a large antral ulcer and edematous mucosa in the duodenal bulb which was biopsied. The duodenoscope could not be advanced past the pylorus because of pyloric narrowing. A follow-up CT scan showed thickening of the gastric antrum and proximal duodenum, a mesenteric haze adjacent to the head of the pancreas, dilatation of the CBD, moderate right-sided hydronephrosis, and bilateral ureteral stents (Figures 2(a) and 2(b)).

### 2.3. Diagnosis

**Figure 3:**
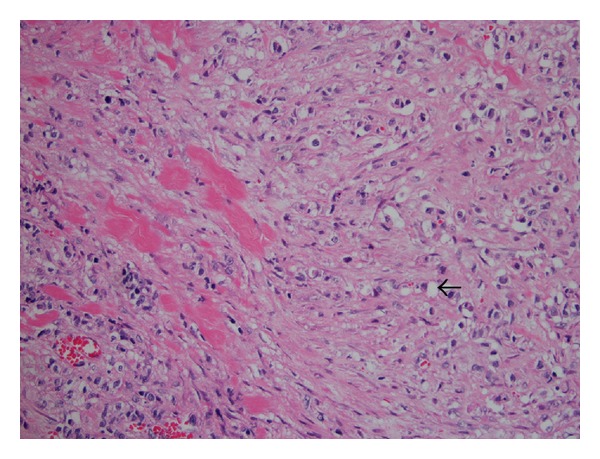
Fibroadipose tissue with infiltrating poorly differentiated adenocarcinoma with signet-ring cell features (arrow), compatible with metastatic adenocarcinoma from known gastric adenocarcinoma.

Preliminary results from biopsies taken during the ERCP showed poorly differentiated carcinoma involving the duodenal mucosa and poorly differentiated gastric adenocarcinoma with signet ring cells. Laparoscopic surgery was recommended by the oncology consultants for diagnosis, staging, and palliative treatment. The procedure was converted to an exploratory laparotomy, colonoscopy, J-feeding tube placement, cholecystostomy, ileostomy, and the placement of a right-sided pleural drain. He developed bilateral deep vein thromboses after the procedure and respiratory distress, requiring reintubation. Inferior vena cava (IVC) filter placement failed because of a narrow IVC, likely due to external compression. Colonoscopy revealed a circumferential rectosigmoid mass extending 15 cm from the anal verge. Laparotomy revealed frozen peritoneal carcinomatosis, ascites, and frozen ascending and descending colon. The stomach was frozen as well, and an end-ileostomy was done in view of impending small bowel obstruction. A pathology slide from the abdominal wall is shown in Figure 3.

### 2.4. Clinical Followup

His renal function started to worsen after the procedure, with a rising creatinine and decreasing urine output (FeNa was 0.51% at the time of transfer to our institution). This was attributed to hemodynamic renal failure in the face of extensive carcinomatosis and liver failure. A renal scan was done to see if there would be any benefit to percutaneous nephrostomy. The study showed slow uptake in both kidneys without evidence of transit or excretion. The persistent activity in the parenchyma was consistent with ATN. Dialysis was not done after a discussion about the patient's overall prognosis, and the patient's family elected comfort care and refused an autopsy.

## 3. Discussion

There are several teaching points that can be made in this case. Bladder wall thickening can be due to several causes, including acute or chronic infection or inflammation, muscular hypertrophy due to outlet obstruction, benign tumors like hemangioma or neurofibroma, or malignancies like transitional cell carcinoma, squamous cell carcinoma, or leiomyosarcoma [2]. Our patient had a rare presentation of gastric cancer in the form of obstruction at the bladder inlet due to tumor infiltration of the bladder wall. Ureteral obstruction is an occasional complication in patients with gastric cancer. When it occurs, it is usually due to one of three conditions: (1) direct extension from the primary site, peritoneal deposit, or lymph node metastasis, (2) retroperitoneal fibrosis, or (3) metastases to the ureters [1]. One review in the Japanese literature describes 24 cases of gastric cancer associated with obstructive uropathy over a five-year period, giving an insight into the frequency of the complication. For an unspecified number of the patients described, back pain and renal failure were the first symptoms of the gastric cancer [3]. A few patients had CT findings of ring-like appearance of the ureters or a thickened wall of renal pelvis or soft-tissue mass directly extending into the renal sinus. Another study from Taiwan reported 17 patients with gastric cancer having obstructive uropathy over a 12-year period [4]. In 3 of the 17 cases, the patients initially presented with back pain and renal failure secondary to obstructive uropathy. All the 17 patients had peritoneal carcinomatosis, and most had either para-aortic lymph nodes or pelvic metastases. Four of the seventeen patients had ureteral obstruction due to metastases invading the bladder. 

Retroperitoneal fibrosis has many malignant causes, including gastric cancer, and can cause bilateral ureteral stenosis and renal failure. In one case, a sixty-two-year-old Japanese female who initially presented with fever, back pain, and renal failure [5]. Imaging revealed bilateral hydronephrosis without any visible obstructing lesion. An endoscopy was performed, as she had been experiencing dysphagia for the previous three months, and demonstrated an ulcer. A biopsy revealed signet-ring cell carcinoma. She had a total gastrectomy and was found to have a poorly differentiated adenocarcinoma invading the entire gastric wall, esophagus, duodenum, and regional lymph nodes. Prominent hard fibrous plaques encased the retroperitoneum, and no surgical treatment could be done to relieve the compression of the ureters. That patient died of renal failure after the operation [5]. We have come across two cases in the French literature describing retroperitoneal fibrosis induced renal failure in cases of gastric cancer [6, 7]. We have also come across a case of bilateral hydronephrosis caused by fibroepithelial polyps in a patient with gastric cancer [8] and also by bilateral ureteral leiomyoma [9]. Retrospective analysis of the scans at our institution did not reveal any retroperitoneal fibrosis or any external compression of the ureters. The tumor in the sigmoid colon was not visualized on imaging. It was more than one month and a second hospitalization with jaundice and a gastrointestinal bleed before the patient had an endoscopy and was diagnosed with gastric cancer. By this time, due to the rapid deterioration in clinical condition, the patient was not a candidate for chemotherapy. It is unclear whether the patient's clinical outcome would have been different had he been diagnosed with gastric cancer during the first admission. In our patient's case, an argument for an urgent endoscopy could have been made, as he had an anemia in addition to his bilateral hydronephrosis. We have come across a case in the Japanese literature describing a 430-year-old woman presenting with advanced scirrhous gastric cancer with left supraclavicular lymph node metastases, massive ascites, rectal stenosis, and bilateral hydronephrosis due to peritoneal metastases [10]. This patient was treated with bilateral ureteral stents and combination chemotherapy with paclitaxel and TS-1. She had 6 cycles of combination chemotherapy and responded well at the time of the case report. Radiographic and endoscopic examinations showed improved compliance in gastric and rectal walls. While we do not know her exact survival, the case offers some hope that such patients who present with such advanced disease may benefit from chemotherapy if diagnosed promptly.

## Figures and Tables

**Table 1:** Laboratory results from first hospitalization (normal values in parentheses).

Hgb 10.2 (14–17.4 g/dL)	Na 136 (135–145 mEq/L)	Lipase 23 (10–195 U/L)	Urine Cr 232 mg/dL (20–370)
Hct 29.9 (40.1–50.4%)	K 5.4 (3.5–5.0 mEq/L)	Alb 3.3 (3.4–4.8 g/dL)	Urine Na 24 mEq/L
WBC 11.6 (4.8–10.8 k/*μ*L)	BUN 58 (8–26 mg/dL)	Prot 5.8 (6.0–8.5 g/dL)	Urine K 45 mEq/L
PLT 208 (150–400 k/*μ*L)	Cr 2.9 (0.5–1.5 mg/dL)	ALT 20 (5–40 U/L)	Urine Cl 13.3 mEq/L
TBILI 0.5 (0.2–1.2 mg/dL)	HC0_3_ 18 (24–30 mEq/L)	AST 16 (5–40 U/L)	
DBILI 0.3 (0.1–0.3 mg/dL)	Cl 105 (98–108 mEq/L)		

Urine Analysis: 21 WBCs, 18 RBCs, 100 protein, unremarkable cytology.

**Table 2:** Admission laboratory results from second hospitalization (normal values in parentheses).

Hgb 8.7 (14–17.4 g/dl)	Na 140 (135–145 mEq/L)	Lipase 577 (10–195 U/L)	Urine Na 24 mEq/L
Hct 26 (40.1–50.4%)	K 4.2 (3.5–5.0 mEq/L)	Alb 3.3 (3.4–4.8 g/dL)	Urine K 45 mEq/L
WBC 8.0 (4.8–10.8 k/*μ*l)	BUN 12 (8–26 mg/dL)	Prot 6.5 (6.0–8.5 g/dL)	Urine Cl 13.3 mEq/L
PLT 200 (150–400 k/*μ*L)	Cr 2.1 (0.5–1.5 mg.dL)	ALT 488 (5–40 U/L)	Urine Cr 232 (20–370 mg/dL)
TBILI 4.2 (0.2–1.2 mg/dL)	HCO3 18 (24–30 mEq/L)	AST 377 (5–40 U/L)	
DBILI 0.8 (0.1–0.3 mg/dL)	Cl 188 (98–108 mEq/L)	ALP 1046 (53–128 U/L)	

Urine analysis: WCC 18, RBC 100, positive for bacteria, eosinophils, and muddy brown casts.
